# Supplementary material for: Topologically enhanced harmonic generation in a nonlinear transmission line metamaterial
Source: Nat Commun. 2019 Mar 7;10:1102. doi: 10.1038/s41467-019-08966-9 (PMC6405745; doi:10.1038/s41467-019-08966-9)
Supplement: Supplementary file 1 — Supplementary Information [file 41467_2019_8966_MOESM1_ESM.pdf]

Supplementary Information for:
Topologically Enhanced Harmonic Generation in a Nonlinear Transmission Line Metamaterial

You Wang, Li-Jun Lang, Ching Hua Lee, Baile Zhang, and Y. D. Chong

Supplementary Note 1 : Linear circuit

Consider the circuit shown in Supplementary Fig. 1a below. This is the same as Fig. 1a of the main text, but with the complex voltage variables relabeled for convenience. The band diagram, plotted in Supplementary Fig. 1b, shows that the upper band exhibits negative dispersion, regardless of the value of α. On unit cell n, the voltages on the two sites are v_n^a (to the right of the C_a capacitor) and v_n^b (to the right of the C_b capacitor). Also, we let i_n^a (i_n^b) denote the current through the inductor to the right of the C_a (C_b) capacitor.

Let C_a, C_b, and L be constants, and take a harmonic mode with angular frequency ω. For $n > 1$, we apply Kirchhoff's laws to the inductors and capacitors, with the $\exp(i\omega t)$ phasor convention, and obtain

$$i\omega L i_n^a + v_n^a = 0 \tag{1}$$

$$i\omega L i_n^b + v_n^b = 0 \tag{2}$$

$$-i\omega\, C_a \left(v_n^a - v_{n-1}^b\right) + i_n^a + i\omega\, C_b \left(v_n^b - v_n^a\right) = 0 \tag{3}$$

$$-i\omega\, C_b \left(v_n^b - v_n^a\right) + i_n^b + i\omega\, C_a \left(v_{n+1}^a - v_n^b\right) = 0. \tag{4}$$

Combining these to eliminate i_n^a and i_n^b yields the following pair of coupled equations:

$$v_{n-1}^b - \frac{1}{\alpha} v_n^a + \frac{1}{\alpha} v_n^b = \left(1 - \Omega^2\right) v_n^a \tag{5}$$

$$\frac{1}{\alpha} v_n^a - \frac{1}{\alpha} v_n^b + v_{n+1}^a = \left(1 - \Omega^2\right) v_n^b, \tag{6}$$

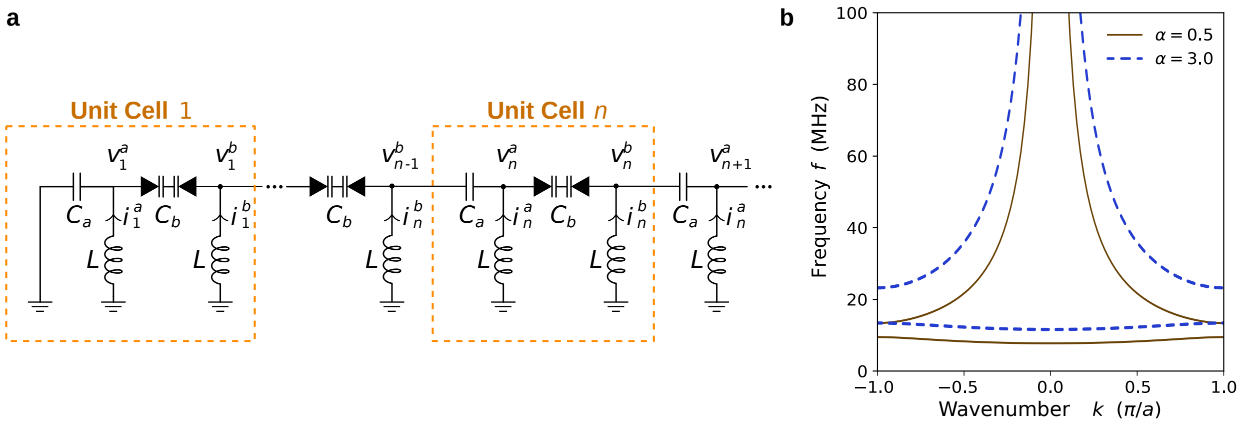

Supplementary figure 1. **a** Schematic of the left-handed transmission line circuit. **b** Band diagram for the infinite lattice. Results are shown for $\alpha = 0.5$ (solid curves) and 3.0 (dashes).

where $\Omega^2 \equiv \omega_a^2/\omega^2$, $\omega_a \equiv 1/\sqrt{LC_a}$ and $\alpha \equiv C_a/C_b$.

Suppose we close the circuit by grounding the leftmost and rightmost sites. Consider the left edge (the right edge is handled similarly). There, the Kirchhoff equations simplify to

$$i\omega L i_1^a + v_n^a = 0 \tag{7}$$

$$-i\omega\, C_a\, v_1^a + i_1^a + i\omega\, C_b \left(v_1^b - v_1^a\right) = 0, \tag{8}$$

resulting in the boundary equation

$$-\frac{1}{\alpha} v_1^a + \frac{1}{\alpha} v_1^b = \left(1 - \Omega^2\right) v_1^a. \tag{9}$$

Hence, we arrive at the modified SSH problem discussed in the main text:

$$\begin{pmatrix} -\frac{1}{\alpha} & \frac{1}{\alpha} & & & \\ \frac{1}{\alpha} & -\frac{1}{\alpha} & 1 & & \\ & 1 & -\frac{1}{\alpha} & \frac{1}{\alpha} & \\ & & \frac{1}{\alpha} & -\frac{1}{\alpha} & \ddots \\ & & & \ddots & \ddots \end{pmatrix} \begin{pmatrix} v_1^a \\ v_1^b \\ v_2^a \\ v_2^b \\ \vdots \end{pmatrix} = \left(1 - \Omega^2\right) \begin{pmatrix} v_1^a \\ v_1^b \\ v_2^a \\ v_2^b \\ \vdots \end{pmatrix}. \tag{10}$$

This is the configuration referred to in the main text as the "nontrivial lattice". The "trivial lattice" can be described by removing the first row and column of the matrix. In either case, the edges of the band gap are

$$\omega_\pm = \left\{ \sqrt{\frac{\alpha}{2}}\omega_a \quad \text{or} \quad \frac{\omega_a}{\sqrt{2}} \right\}, \tag{11}$$

and the angular frequency of the edge state is

$$\omega_{\text{es}} = \sqrt{\alpha/(1+\alpha)}\, \omega_a. \tag{12}$$

Next, we consider the response of the circuit to a harmonic voltage source. Instead of grounding the left edge, we apply an input voltage of amplitude V_{in} and frequency ω. Then equation (9) is replaced by

$$V_{\text{in}} - \frac{1}{\alpha} v_1^a + \frac{1}{\alpha} v_1^b = \left(1 - \Omega^2\right) v_1^a, \tag{13}$$

and the eigenvalue equation (10) is replaced by an inhomogenous equation.

Numerical solutions for this problem are shown in Supplemetary Fig. 2. Supplementary Fig. 2a–b shows that the voltage amplitude in the nontrivial lattice is resonantly enhanced

when ω matches ω_{es}. The trivial lattice, on the other hand, does not exhibit a resonant enhancement. However, when we plot the voltage distributions, they have the same decay constant, as shown in Supplementary Fig. 2c. This is due to the fact that they have the same *bulk* Hamiltonians.

Supplementary Fig. 2d shows the modal intensity (the sum of squared voltage amplitudes over the lattice) at resonance, versus the α parameter, for a nontrivial lattice. In this plot, the input frequency for each value of α is adjusted to the edge state frequency given by equation (12). With increasing α, the bandgap becomes larger (i.e., the lattice moves

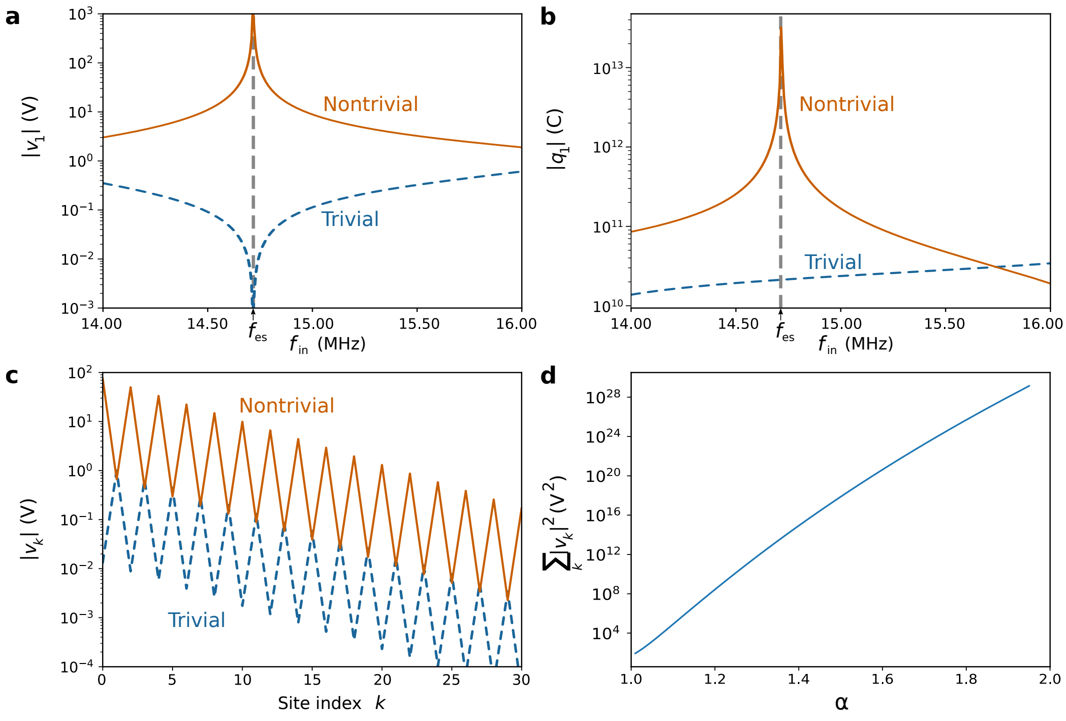

Supplementary figure 2. Response of the linear circuit to an external voltage, calculated numerically from the circuit equations with the parameters $f_a = 19\,\mathrm{MHz}$ and $C_a = 47\,\mathrm{pF}$. **a**–**b** Resonant response of (**a**) the amplitude of the voltage on the first site (to the right of the leftmost capacitor), and (**b**) the amplitude of the stored charge on the first (leftmost) capacitor, versus source frequency $f_{\mathrm{in}} = \omega/2\pi$, for $\alpha = 1.5$. The resonance frequency predicted by equation (12) is indicated by the horizontal dashed line. **c** Spatial distribution of the voltage ampltidues for $f_{\mathrm{in}} = 14.75\,\mathrm{MHz}$ and $\alpha = 1.5$. **d** Intensity of the nontrivial lattice's resonant mode, as given by the value of $\sum_k |v_k|^2$ (where $|v_k|$ is the voltage amplitude at site k) at resonance, versus the α parameter. For each value of α, the input voltage has amplitude $1\,\mathrm{V}$ and frequency given by equation (12).

deeper into the topologically nontrivial phase); accordingly, the edge state is more strongly confined, and responds more strongly to the resonant excitation. The modal intensity scales exponentially with the bandgap size.

Supplementary Note 2 : Circuit equations for nonlinear circuit

We seek a set of time-domain equations for the circuit's nonlinear regime, where the B capacitors are nonlinear. Let $q_n^a(t)$ and $q_n^b(t)$ denote the charges stored in capacitors A and B, respectively, on site n. These obey

$$q_n^a(t) = C_a \Big[v_n^a(t) - v_{n-1}^b(t)\Big], \tag{14}$$

$$q_n^b(t) = C_{bn}(t) \Big[v_n^b(t) - v_n^a(t)\Big]. \tag{15}$$

Here, $C_{bn}(t)$ is the value of the nonlinear B capacitance in unit cell n. Using Kirchhoff's laws, we can derive several additional equations. The time-dependent voltage-current relations on the inductors are

$$v_n^a = -L\frac{di_n^a}{dt}, \tag{16}$$

$$v_n^b = -L\frac{di_n^b}{dt}. \tag{17}$$

The current-charge relations on the capacitors, with the assumptions of current conservation and zero net charge, give

$$\frac{dq_n^a}{dt} - \frac{dq_n^b}{dt} = i_n^a \tag{18}$$

$$\frac{dq_n^b}{dt} - \frac{dq_{n+1}^a}{dt} = i_n^b. \tag{19}$$

By combining Eqs. (14)–(19), we can eliminate the $q^{a/b}$ and $i^{a/b}$ variables, resulting in the following pair of time-domain circuit equations expressed in terms of the $v^{a/b}$ variables:

$$-\frac{d^2}{dt^2}\left[v_n^a - v_{n-1}^b - \frac{1}{\alpha_n}\left(v_n^b - v_n^a\right)\right] = \omega_a^2\, v_n^a(t) \tag{20}$$

$$-\frac{d^2}{dt^2}\left[v_n^b - v_{n+1}^a + \frac{1}{\alpha_n}\left(v_n^b - v_n^a\right)\right] = \omega_a^2\, v_n^b(t). \tag{21}$$

Here,

$$\alpha_n(t) \equiv \frac{C_a}{C_{bn}(t)} \tag{22}$$

is the nonlinear capacitance ratio at site n.

It is convenient to re-cast Eqs. (20)–(21) in terms of the variables

$$u_n = v_n^b + v_n^a \tag{23}$$

$$w_n = v_n^b - v_n^a. \tag{24}$$

Then

$$-\frac{d^2}{dt^2}\left[-\frac{1}{2}u_{n+1} + u_n - \frac{1}{2}u_{n-1} + \frac{w_{n+1} - w_{n-1}}{2}\right] = \omega_a^2 u_n \tag{25}$$

$$-\frac{d^2}{dt^2}\left[-\frac{u_{n+1} - u_{n-1}}{2} + \frac{w_{n+1} + w_{n-1}}{2} + \left(1 + \frac{2}{\alpha_n(t)}\right) w_n\right] = \omega_a^2 w_n. \tag{26}$$

Supplementary Note 3 : Harmonic decomposition and nonlinearity model

If a harmonic signal is injected into the nonlinear circuit, higher harmonics are generated. Due to the symmetric C-V curve of the nonlinear capacitors, even-order harmonics are suppressed.

Let ω denote the frequency of the first harmonic. We will decompose the voltage variables in the following way:

$$u_n(t) \approx \sum_{m=1,3,5,\cdots} (-1)^n \, U_n^m \, e^{im\omega t} \;\; + \text{ c.c.} \tag{27}$$

$$w_n(t) \approx \sum_{m=1,3,5,\cdots} (-1)^n \, W_n^m \, e^{im\omega t} \; + \text{ c.c.} \tag{28}$$

On the right hand sides, the integer superscripts $\{1, 3, 5, \dots\}$ denote the harmonic index. The factor of $(-1)^n$ is for later convenience; we expect the first harmonic mode to behave like an SSH edge state, which is characterized by alternating signs on adjacent unit cells (another way of saying this is that the band gap of the bulk SSH model is narrowest at the Brillouin zone boundary, $k = \pm\pi/h$, where h is the lattice constant), and this factor ensures that the U_n^1 and W_n^1 variables act as smooth envelopes with the sign alternation taken out.

We now have to substitute the ansatz (27)–(28) into Eqs. (25)–(26). First, consider equation (25), which is easy to deal with since it is linear. Matching the individual harmonics, we obtain

$$\frac{1}{2}U_{n+1}^1 + U_n^1 + \frac{1}{2}U_{n-1}^1 - \frac{1}{2}\left(W_{n+1}^1 - W_{n-1}^1\right) = \Omega_1^2 \, U_n^1, \tag{29}$$

where

$$\Omega_m^2 \equiv \frac{\omega_a^2}{m^2\omega^2}. \tag{30}$$

Next, consider the nonlinear equation (26). The main complication here is the term involving

$$\frac{w_n(t)}{\alpha_n(t)}. \tag{31}$$

The time variation of $\alpha_n(t)$ gives rise to two classes of effects: (i) self-phase modulation and cross-phase modulation, which alter the effective value of α_n "seen" by each given harmonic, and (ii) frequency-mixing processes, which couple the dynamical equations for the different harmonics. For now, let us try to pick out the contributions to category (i), neglecting (ii).

In our experiment, each nonlinear capacitor consists of a pair of back-to-back varactors. The nonlinear capacitance ratio α_n was defined in equation (22). Let us make the assumption that

$$\alpha_n(t) \;\approx\; A + B\left[v_n^b(t) - v_n^a(t)\right]^2 \;=\; A + B\left[w_n(t)\right]^2. \tag{32}$$

Here, A is the capacitance ratio in the linear limit and $B > 0$ is a Kerr-like parameter determining the strength of the lowest-order nonvanishing (cubic) nonlinearity. To obtain values for A and B, we use the manufacturer-supplied capacitance-voltage curve for the individual varactors to calculate α and the bias voltage ΔV for a pair of back-to-back varactors. We then perform a linear least-squares fit of α versus ΔV^2, using the subset of data points with voltage biases $\Delta V \leq 1\,\mathrm{V}$. The fitted parameters are $A = 1.32$ and $B = 0.51\,\mathrm{V}^{-2}$, and the fit is shown in Fig. 1c of the main text.

In (31), we can take the approximation of replacing $\alpha_n(t)$ with its time-independent part,

$$\langle\alpha_n\rangle \approx A + 2B \sum_{m=1,3,\ldots} \left|W_n^m\right|^2. \tag{33}$$

For each harmonic m, this would then give rise to a term

$$\propto \frac{W_n^m}{\langle\alpha_n\rangle}\, e^{im\omega t}, \tag{34}$$

with $\langle\alpha_n\rangle$ now playing the role of an "effective" α parameter.

This approximation does not capture all possible self-phase and cross-phase modulation terms. This can be seen in the low-intensity limit, where we can Taylor expand $1/\alpha_n(t)$ in the W_n^m variables; in this expansion, there will be non-constant terms like $W_n^m (W_n^{m'})^* e^{i(m-m')\omega t}$, which couples to the $W_n^{m'} e^{im'\omega t}$ harmonic term from $w_n(t)$ to yield a term proportional to

$W_n^m\, e^{im\omega t}$, and hence contributing to the self-phase or cross-phase modulation. We will not undertake a rigorous analysis of these terms, since the Taylor expansion is invalid anyway when the intensities are not small. Instead, our take-home message is as follows:

1. Each harmonic contributes to the effective value of α in direct proportion to its local intensity, like in equation (33).

2. However, the precise numerical factor need not be exactly the same as in equation (33).

Based on this approximation, we can now deal with the nonlinear equation (26), which simplifies to

$$\frac{U_{n+1}^1 - U_{n-1}^1}{2} - \frac{W_{n+1}^1 + W_{n-1}^1}{2} + \left(1 + \frac{2}{\langle \alpha_n \rangle}\right) W_n^1 = \Omega_1^2\, W_n^1. \tag{35}$$

Supplementary Note 4 : Localized and traveling-wave solutions

Let us consider the case where $\langle \alpha_n \rangle$ is approximately constant in space, and look for solutions of the form

$$U_n^m = U_m e^{ik_m n}, \quad W_n^m = W_m e^{ik_m n}. \tag{36}$$

These are traveling-wave solutions if k_m is real, and exponentially localized solutions if k_m is complex. Substituting this into Eqs. (29) and (35) gives

$$\begin{pmatrix} 1 + \cos k_m & -i \sin k_m \\ i \sin k_m & 1 + \frac{2}{\langle \alpha \rangle} - \cos k_m \end{pmatrix} \begin{pmatrix} U_m \\ W_m \end{pmatrix} = \Omega_m^2 \begin{pmatrix} U_m \\ W_m \end{pmatrix}. \tag{37}$$

Solving the characteristic equation gives

$$\cos k_m = \Big(1 + \langle \alpha \rangle\Big)\Omega_m^2 - \Big(1 + \frac{\langle \alpha \rangle}{2}\Omega_m^4\Big). \tag{38}$$

We can then easily show that, for $\langle \alpha \rangle > 1$, the domains over which the right-hand side has magnitude smaller than unity (i.e., k_m is real) are:

$$\Omega_m^2 < 2/\langle \alpha \rangle \tag{39}$$

$$2 < \Omega_m^2 < 2(1 + 1/\langle \alpha \rangle). \tag{40}$$

For $m = 1$, this corresponds exactly to the bands shown in Fig. 1d–e of the main text. In particular, within the band gap between $\omega_a/\sqrt{2}$ and $\sqrt{\alpha/2}\,\omega_a$, the right-hand side is larger than unity and hence k_1 is imaginary, in complete agreement with the linear analysis.

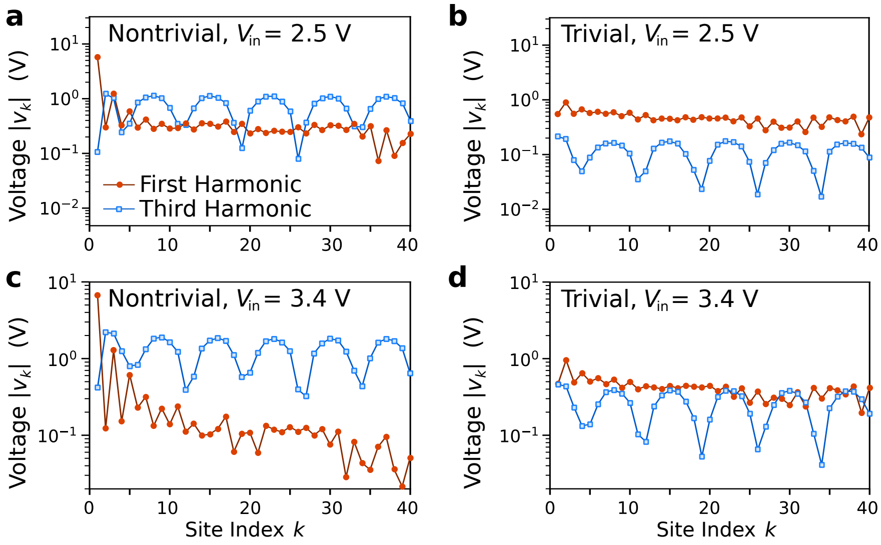

Supplementary figure 3. On-site voltage amplitudes for the first-harmonic signal (orange circles) and third-harmonic signal (blue squares). The frequency of the input is $f_{\text{in}} = 16.4\,\text{MHz}$. **a**, **b** Nontrivial and trivial lattices with $V_{\text{in}} = 2.5\,\text{V}$. **c**, **d** Nontrivial and trivial lattices with $V_{\text{in}} = 3.4\,\text{V}$.

For the higher-harmonic modes, (39) is satisfied easily. For example, for the third harmonic, we require

$$\omega^2 > \frac{\langle\alpha\rangle}{18}\omega_a^2. \tag{41}$$

For operating frequencies below the linear-regime band gap, $\omega < \sqrt{A/2}\,\omega_a$, this is satisfied for

$$\alpha < 9A, \tag{42}$$

which is well within the regime considered in this experiment. This analysis thus confirms that the nonlinear circuit is capable of supporting traveling-wave higher-harmonic solutions.

Supplementary Note 5 : Simulated voltage profiles

We performed simulations of the nonlinear circuit using the circuit simulation software SPICE. The simulations reproduce the basic features of the experimental results, though the results are not in exact agreement, probably due to imperfections in the circuit components.

Supplementary Fig. 3 shows the on-site voltage amplitudes for the first- and third-harmonic signals. These are extracted from the simulation results in a manner similar to the experiment: after the simulation reaches steady-state, we take a time-dependent sample,

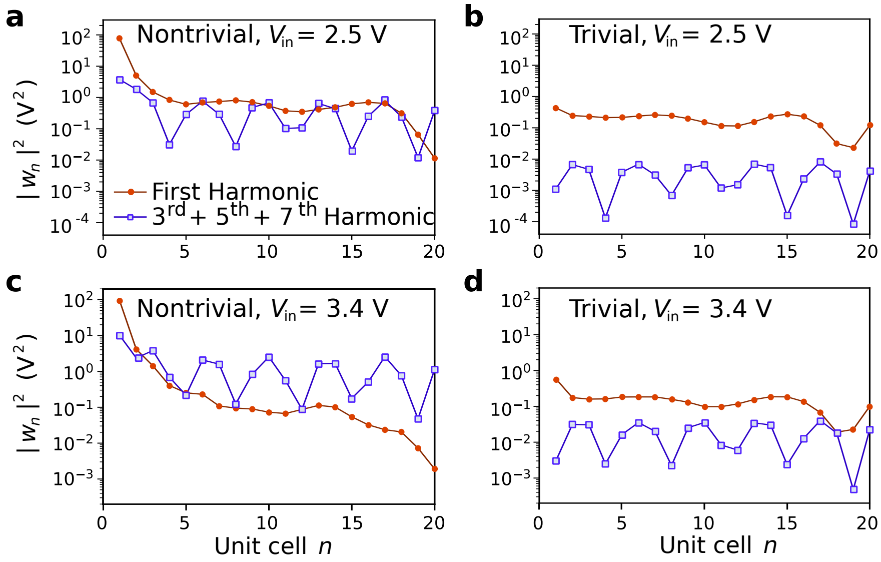

Supplementary figure 4. Squared bias voltage amplitudes on the nonlinear capacitors, for the first-harmonic signal (orange circles) and higher-harmonic signals (purple squares). The frequency of the input is $f_{\text{in}} = 16.4\,\text{MHz}$. The higher-harmonic data is obtained by summing over the third, fifth, and seventh harmonic data (further harmonics are negligible). **a**, **b** Nontrivial and trivial lattices with $V_{\text{in}} = 2.5\,\text{V}$. **c**, **d** Nontrivial and trivial lattices with $V_{\text{in}} = 3.4\,\text{V}$.

Fourier transform, and extract the peak heights. To obtain simulation results matching the experimental results shown in Fig. 2a–b of the main text, we find that it is necessary to apply a higher input voltage amplitude than in the experiment, $V_{\text{in}} \approx 3.4\,\text{V}$. The results are shown in Supplementary Fig. 3c–d. Similar to the experiment, the first-harmonic mode in the nontrivial lattice decays away from the edge, reaching values much lower than in the trivial lattice.

Supplementary Fig. 4 shows the bias voltage amplitudes on the nonlinear capacitors, which were not measured in the experiment. As discussed in Supplementary Note 3, the bias voltages determine the effective value of the nonlinear α parameter. To obtain this data from the simulations, we extract the time-dependent bias voltage samples (i.e., the time-dependent voltages between the ports of the nonlinear capacitors, denoted by $w_n(t)$ in Supplementary Note 2), Fourier transform, and extract the peak heights; this yields the components denoted by $|W_n^m|$ in Supplementary Note 3. According to equation (33), the contribution of each harmonic to the effective local α is proportional to $|W_n^m|^2$. Supplementary Fig. 4 shows a comparison between the first-harmonic contribution (orange circles) and the higher-harmonic

contributions (purple squares). In particular, in Supplementary Fig. 4c, which correspond to the voltage plot of Supplementary Fig. 3c, the higher-harmonic signals are found to increasingly dominate the nonlinearity as we go deeper into the lattice.

Supplementary Note 6 : SPICE simulation results: mid-lattice excitation

If we choose to excite a site in the middle of the circuit, rather than the edge, the behavior depends on the input impedance of the voltage source. First, consider a low-impedance voltage source. In this case, the voltage on the input site is rigidly determined by the voltage source, so this is similar to exciting two independent transmission lines.

If lattice is uniform (i.e., defect free), once we pick an excitation site, the lattice sections to the left and right of the excitation site necessarily have different topological phases. For instance, for $\alpha > 1$, if there is a C_a capacitor to the right of the excitation site, then the section on the right is nontrivial and the section on the left is trivial. Under such circumstances, reasoning from the behavior of the circuit under edge excitation, we expect the higher-harmonic signal to be emitted asymmetrically: a strong higher-harmonic signal should propagate to the topologically nontrivial side, with a weak higher-harmonic signal on the trivial side. This prediction is verified by the SPICE simulation results plotted in Supplementary Fig. 5.

When the input impedance of the voltage source is high, the behavior is less clear-cut, as both the first- and higher-harmonic signals can easily cross the excitation site. In both the topologically trivial and nontrivial lattices, the higher-harmonic modes are in-band; thus, any higher-harmonic signal that is generated can propagate to either the trivial or nontrivial side. At each frequency, the dominant direction of higher-harmonic emission will depend on the availability of circuit modes, based on finite-size effects, in each lattice section.

Supplementary Note 7 : Simulation comparisons with conventional NLTL

Finally, we used SPICE simulations to compare the third harmonic intensity in this circuit to a conventional left-handed NLTL. In the conventional left-handed NLTL, all the linear C_a capacitors are replaced with nonlinear C_b capacitors (i.e., the lattice is no longer dimerized). In the linear limit, the conventional left-handed NLTL with $C_b = 35\,\mathrm{pF}$ is a high-pass filter

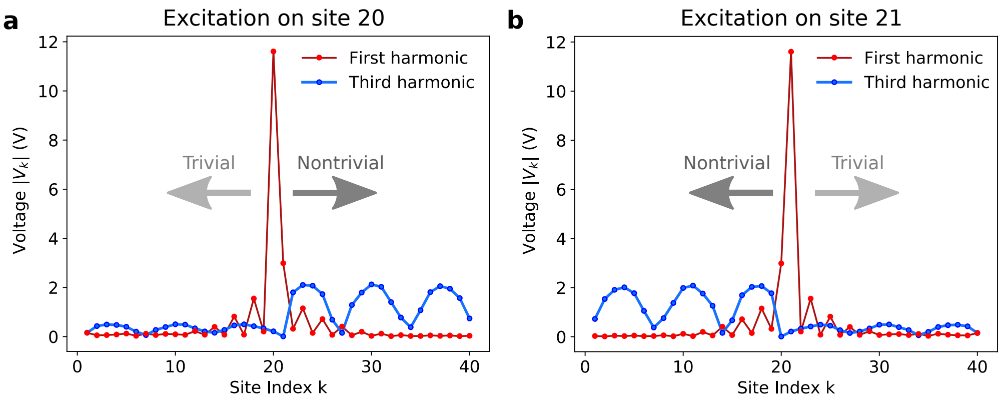

Supplementary figure 5. Voltage amplitude profiles for the first and third harmonic signals, from SPICE simulation on a 40-site NLTL with (**a**) site 20 excited, and (**b**) site 21 excited. The choice of excitation site partitions the lattice into topologically trivial and nontrivial sections. The input signal has voltage amplitude $V_{\text{in}} = 3\,\text{V}$, frequency $f_{\text{in}} = 16\,\text{MHz}$, and input impedance 1Ω. All NLTL simulation parameters are the same as in Section Supplementary Note 5.

with a Bragg cutoff frequency of 11 MHz.

Supplementary Fig. 6 plots the simulation results for the normalized third-harmonic intensity χ (defined in the same way as in the main text), versus the input parameters f_{in} and input voltage V_{in}. The simulation results for the SSH-like lattice, shown in Supplementary Fig. 6a, are similar to the experimental results shown in Fig. 3a of the main text; in particular, the maximum value of χ is $\gtrsim 2.4$, comparable to the experimentally-obtained maximum value $\chi \approx 2.5$. By contrast, Supplementary Fig. 6b shows that the conventional NLTL exhibits no comparable enhancement of the third-harmonic signal intensity, with $\chi < 0.47$ throughout the entire parameter regime we investigated. Hence, the introduction of the topological edge mode has contributed to a five-fold increase in the intensity of the generated third-harmonic signal.

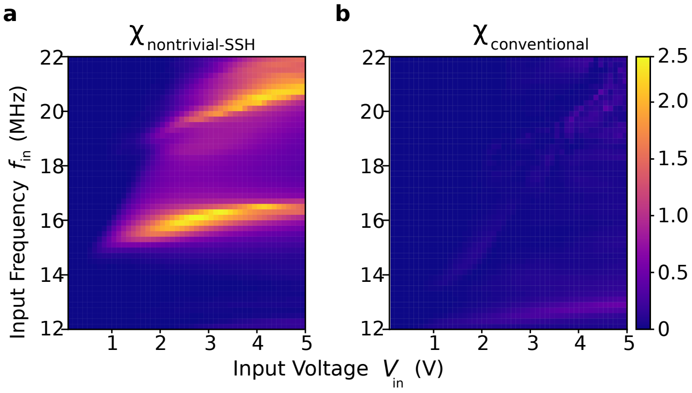

Supplementary figure 6. Plot of the normalized third-harmonic intensity χ versus input frequency f_{in} and input voltage V_{in}, for (**a**) the SSH-like lattice in its topologically nontrivial configuration, and (**b**) a conventional left-handed nonlinear transmission line (NLTL) with identical nonlinear capacitors. The figure of merit χ is defined in the same way as in the main text, as the mean squared third-harmonic amplitude on the first 10 sites relative to the squared input voltage amplitude. In the SSH case, we obtain $\chi \gtrsim 2.4$, which is comparable to the experimental result of $\chi \approx 2.5$ reported in the main text. In the conventional NLTL, we observe $\chi < 0.47$ throughout the parameter range.
